# Severe Insomnia Induced by Isotretinoin Use for the Management of Acne Vulgaris: A Rare Side Effect

**DOI:** 10.7759/cureus.33658

**Published:** 2023-01-11

**Authors:** Sultan H Assiri, Ashwaq K Alosaimi, Emad A Alharbi

**Affiliations:** 1 Department of Dermatology, King Abdulaziz Hospital, Makkah, SAU

**Keywords:** acne, sleep-disorder, depression, isotretinoin, insomnia

## Abstract

Isotretinoin has been widely prescribed for the management of moderate to severe acne vulgaris worldwide. It has been associated with different sleep disorders, mainly sleep apnea and hypersomnia. To our knowledge, only one study has found an association between isotretinoin use and insomnia. In addition, psychiatric disorders, such as psychosis and depression, have been linked to the use of isotretinoin previously. Here, we present a case of a 21-year-old male patient with severe acne vulgaris that underwent a therapeutic course of isotretinoin. During the course of treatment, he developed severe insomnia and depression. By reducing the daily dose, his insomnia was treated, as well as his depression. This case led to the conclusion that isotretinoin is associated with severe insomnia, and the severity of insomnia is dose-dependent.

## Introduction

Isotretinoin has been widely used in the management of moderate to severe acne vulgaris with great results. However, numerous side effects have been associated with its use. The association between isotretinoin and different sleep disorders has been previously established in the literature. In our literature review, we identified a relationship between isotretinoin and increased frequency of sleep apnea [1], hypersomnia [2], and improved night-time sleep [3]. To our knowledge, only one study established the relationship between isotretinoin and insomnia.

Furthermore, the use of isotretinoin for the treatment of acne has been linked to various psychiatric disorders such as depression and psychosis [4,5]. Since insomnia could be the first symptom of psychiatric disorders [4], it is essential to ask susceptible patients using isotretinoin about insomnia and other psychiatric symptoms. Here, we present a case in which a patient develops insomnia shortly after the initiation of isotretinoin for the treatment of acne.

## Case presentation

Mr. H, a 21-year-old male with no prior history of medical or psychiatric issues, presented to us in the Dermatology department with severe inflammatory cystic acne on the face, chest, and back. Due to the failure of previous topical treatments, the severity of the disease, and the presence of acne scars on the face, we started him on isotretinoin 40 mg once per day. The dose was decided based on the severity of the disease and the patient’s weight, 60 kg. Six weeks after the initiation of isotretinoin 40 mg, the patient developed severe insomnia. After five months of treatment, he started reporting suffering from this severe insomnia. Since the start of isotretinoin 40 mg, the patient slept only for two to three hours a day. Two of his cousins had the same problem when they used isotretinoin as well. On top of the fact that he never had any problems with sleeping before, he stated that he could previously sleep for 13-15 hours per day. The patient now had trouble falling asleep. It would take him one to two hours to fall asleep. He did not have any anxiety before sleeping. He tried different ways to help him sleep. He took warm showers, ate heavy meals, drank milk, and took some sleeping medications without any benefits. Moreover, he had trouble maintaining sleep, with frequent waking up in the middle of the night. To measure the severity of his insomnia, we used the ‘Insomnia Severity Index’ (Appendices). He scored 23 out of 28, indicating severe clinical insomnia. Initially, this lack of sleep led our patient to have low energy and decreased productivity during the day. The consequences were gradually getting worse, resulting in a lack of motivation, depression, social withdrawal, and loss of appetite. As a consequence, he lost 6 kg. Due to the severity of his insomnia, we reduced the dose to 20 mg per day. He took this dose for one month without any benefits regarding his insomnia. In the next follow-up, we reduced the dose further to 10 mg per day. A few days later, the patient’s sleep pattern dramatically improved. He slept for seven to eight hours without interruptions. His quality of life was, as well, improved. He was very satisfied and happy with this improvement. Compared to scoring high (23/28) previously on the Insomnia Severity Index, he now scored 3/28, indicating no clinically significant insomnia. Luckily, the reduction of the dose did not have any negative impact on his acne. Finally, we continued the same dose, 10 mg per day, until the end of the course.

## Discussion

The intention of this report is two-fold. The first intention is to bring attention to the possible relationship between the use of isotretinoin and insomnia as a serious side effect. Second, this report intends to increase awareness of the potential psychiatric risk of using isotretinoin in patients with/without a history of psychiatric disorders such as depression and psychosis.

In the case of our patient, the patient has never had any trouble falling asleep or insomnia before the initiation of isotretinoin for his severe acne. To establish whether isotretinoin was the culprit for his insomnia, we reduced the dose and re-evaluated the patient monthly. There was a significant improvement in his insomnia, as measured by ISI. Our finding that insomnia could be caused by isotretinoin is in line with other findings from a retrospective cohort study conducted by Gupta et al. in 2020 [4]. They extracted data from the US Food and Drug Administration (FDA adverse event reporting system (FAERS). Out of 218,594 individual safety reports regarding adverse events of isotretinoin, 1095 concerned insomnia. The correlation between insomnia and isotretinoin was significant (P<.0001) compared with all other treatments for acne. The investigators of the aforementioned study recommended screening young patients using isotretinoin who develop insomnia for mood disorders. Based on their recommendation, we screened our patient for mood disorders.

Furthermore, insomnia has been widely described in the literature as a common symptom in the early stage of mood/psychiatric disorders. In our case, the patient did not have any personal or family history of mood/psychiatric disorders. By taking a thorough history of our patient, no history of psychological or psychiatric symptoms was identified. However, this does not exclude the possibility that his insomnia could be an early symptom of a hidden psychological disorder, as was established by another study [5]. In the study by Suuberg et al. in 2019, one patient with insomnia after two months of starting isotretinoin developed acute psychosis that went into remission after stopping isotretinoin [5]. This points toward the importance of paying extra attention to patients that develop insomnia after the initiation of isotretinoin. Even though our patient did not have any psychological history, it is still of great importance to thoroughly monitor the patient and screen him for psychological/mood disorders if needed.

## Conclusions

The use of isotretinoin for the management of moderate-severe acne vulgaris has been found to be associated with severe insomnia. As a consequence, patients with severe insomnia could develop mood disorders, such as depression and psychosis. Insomnia and depression could be improved by reducing the daily dose. Finally, patients with insomnia after the initiation of isotretinoin should be monitored and screened for mood disorders, as this could be the initial symptom of a drug-induced psychiatric disorder.

## Figures and Tables

**Figure 1:**
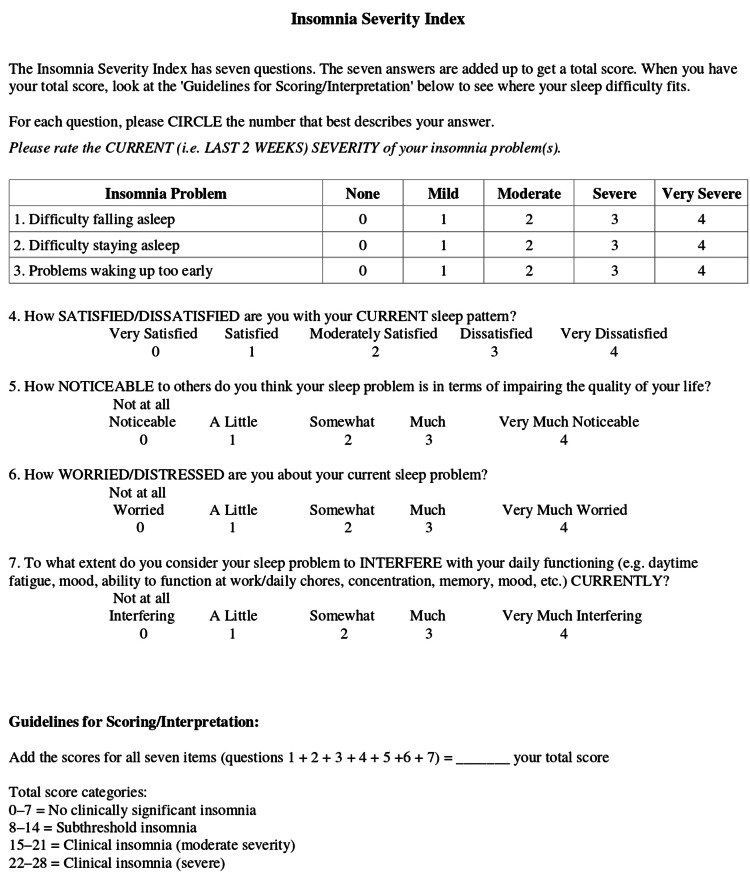
Insomnia Severity Index
